# County-Level Geographic Disparities in Disabilities Among US Adults, 2018

**DOI:** 10.5888/pcd20.230004

**Published:** 2023-05-11

**Authors:** Hua Lu, Yan Wang, Yong Liu, James B. Holt, Catherine A. Okoro, Xingyou Zhang, Qing C. Zhang, Kurt J. Greenlund

**Affiliations:** 1 Division of Population Health, National Center for Chronic Disease Prevention and Health Promotion, Centers for Disease Control and Prevention, Atlanta, Georgia; 2 Division of Human Development and Disability, National Center on Birth Defects and Developmental Disabilities, Centers for Disease Control and Prevention, Atlanta, Georgia; 3 Office of Compensation and Working Conditions, US Bureau of Labor Statistics, Washington, District of Columbia

## Abstract

**Introduction:**

Local data are increasingly needed for public health practice. County-level data on disabilities can be a valuable complement to existing estimates of disabilities. The objective of this study was to describe the county-level prevalence of disabilities among US adults and identify geographic clusters of counties with a higher or lower prevalence of disabilities.

**Methods:**

We applied a multilevel logistic regression and poststratification approach to geocoded 2018 Behavioral Risk Factor Surveillance System data, Census 2018 county-level population estimates, and American Community Survey 2014–2018 poverty estimates to generate county-level estimates for 6 functional disabilities and any disability type. We used cluster-outlier spatial statistical methods to identify clustered counties.

**Results:**

Among 3,142 counties, median estimated prevalence was 29.5% for any disability and differed by type: hearing (8.0%), vision (4.9%), cognition (11.5%), mobility (14.9%), self-care (3.7%), and independent living (7.2%). The spatial autocorrelation statistic, Moran’s *I*, was 0.70 for any disability and 0.60 or greater for all 6 types of disability, indicating that disabilities were highly clustered at the county level. We observed similar spatial cluster patterns in all disability types except hearing disability.

**Conclusion:**

The results suggest substantial differences in disability prevalence across US counties. These data, heretofore unavailable from a health survey, may help with planning programs at the county level to improve the quality of life for people with disabilities.

**Summary**

**What is already known on this topic?**

Approximately 67.5 million (26.0%) US adults have at least 1 of 6 disability types: serious difficulty with hearing, vision, cognition, or mobility or any difficulty with self-care or independent living.

**What is added by this report?**

We assessed differences in the county-level prevalence of these 6 types of disabilities and identified county-level geographic clusters of disability prevalence across the US.

**What are the implications for public health practice?**

We found substantial differences among US counties; these data can help disability-related programs to plan at the county level to improve the quality of life for people with disabilities.

## Introduction

In 2018, about 26.0% of US adults (67.5 million) had at least 1 of 6 disability types (ie, serious difficulty with hearing, vision, cognition, or mobility; any difficulty with self-care or independent living) (1), accounting for 36% of national health care spending (2). Compared with people living without disabilities, people with disabilities need more health care and support to address functional limitations and maintain active participation in their communities (3). The prevalence of disabilities varies by race and ethnicity, sex, socioeconomic status, and geographic region (1). Disability is more common among women, older adults, American Indians and Alaska Natives, adults living below the federal poverty level, and adults living in the southern region of the US (4). In 2018, the most prevalent disability was related to mobility, followed by cognition, hearing, independent living, vision, and self-care in the US (5).

Timely information on the prevalence of disabilities at the local level is essential for local governments and health planners to address the needs of people with disabilities such as health care, transportation, and other services. To date, no study has used national health survey data to describe the county-level prevalence of these 6 disabilities. We previously used the Behavioral Risk Factor Surveillance System (BRFSS), a health survey conducted in all states and most US territories, to produce model-based small-area estimates of chronic disease–related measures at the substate level (6). In this study, we estimated the county-level prevalence of disabilities among US adults and identified county-level geographic clusters of the prevalence of disability.

## Methods

BRFSS is an annual state-based health-related telephone (landline and cell phone) survey conducted by each state and the District of Columbia, with assistance from the Centers for Disease Control and Prevention (CDC) (7). Information on chronic diseases, health risk behaviors, use of preventive services, and sociodemographic characteristics is collected among civilian, noninstitutionalized adults aged 18 years or older. BRFSS has included 5 of 6 disability questions (except hearing) since 2013 and all 6 questions since 2016 and is an essential source of state-level health information on people with disabilities (1,7). We estimated the county-level prevalence of the 6 disability types and any disability by using 2018 BRFSS data and a model-based approach, which were consistent with the CDC state-level disability data system (1). We analyzed restricted 2018 BRFSS data with county Federal Information Procesing Standards codes, which we obtained through a data-use agreement. In 2018, 430,949 respondents in the 50 states and the District of Columbia provided complete information. The state median response rate was 49.9% (8).

### Definition of disability types and any disability

In 2018, BRFSS used the US Department of Health and Human Services (9) 6-item set of questions to identify disability status in hearing, vision, cognition, mobility, self-care, and independent living (10). Respondents were identified as having a specific disability if they answered yes to the following questions: 1) Hearing: “Are you deaf or do you have serious difficulty hearing?”; 2) Vision: “Are you blind or do you have serious difficulty seeing, even when wearing glasses?”; 3) Cognition: “Because of a physical, mental, or emotional condition, do you have serious difficulty concentrating, remembering or making decisions?”; 4) Mobility: “Do you have serious difficulty walking or climbing stairs?”; 5) Self-care: “Do you have difficulty dressing or bathing?”; and 6) Independent living: “Because of physical, mental or emotional conditions, do you have difficulty doing errands alone such as visiting a doctor’s office or shopping?” Respondents who answered yes to at least 1 disability question were categorized as having any disability. People were identified as having no disability if they responded no to all 6 questions. We excluded from analysis responses recorded as “don’t know or not sure,” “refused,” and “not asked or missing” (excluded from 430,949 responses were hearing, 10,522; vision, 11,024; cognition, 13,372; mobility, 12,933; self-care, 12,717; and independent living, 14,103).

### Small-area estimation analysis

A multilevel regression and poststratification (MRP) approach used in CDC’s PLACES project (www.cdc.gov/PLACES) was applied to generate county-level estimates for the 7 measures (6 disabilities and any disability) (6,11–14). The model included the following individual-level variables: sex (male or female), 13 age groups (18–24, 25–29, 30–34, 35–39, 40–44, 45–49, 50–54, 55–59, 60–64, 65–69, 70–74, 75–79, or ≥80 y), and 8 race and ethnicity groups (Hispanic, non-Hispanic American Indian or Alaska Native, non-Hispanic Asian, non-Hispanic Black, non-Hispanic Native Hawaiian or Other Pacific Islander, non-Hispanic White, non-Hispanic other single race, and non-Hispanic ≥2 races) from the 2018 BRFSS; the county-level percentage of population under 150% of the federal poverty level from the 2014–2018 Amercian Community Survey (ACS) 5-year data (15); and state- and county-level random effects. We then applied the model’s estimated parameters for both fixed effects and random effects to the US Census Bureau’s 2018 county-level population estimates by sex, age, and race and ethnicity (total 208 subpopulation groups) (Vintage 2018) (16) to calculate the predicted probability of each disability and of any disability for each of 208 subpopulation groups by county. The county-level predicted population count with disability was the sum of all 208 subpopulation group counts within a county multiplied by their corresponding predicted probabilities of disability; the county-level disability prevalence estimate was the ratio of the predicted county-level population count with a disability and the corresponding county-level population. We used Monte Carlo simulation to generate 1,000 samples of model parameters to account for the variation of the point prevalence estimates of disability; thus, each county had 1,000 estimated prevalences. We summarized the final estimates for each disability measure as the mean of the 1,000 samples. We calculated median, IQR, and range to show the distributions of county-level estimates among all 3,142 counties.The county-level estimates were also analyzed by urban–rural status (4 metropolitan [large central metro, large fringe metro, medium metro, and small metro counties] and 2 nonmetropolitan [micropolitan and noncore counties]) based on the National Center for Health Statistics’ 2013 urban–rural classification scheme for counties (17,18). We used SAS version 9.4 (SAS Institute Inc) for all analyses.

### Spatial cluster-outlier analysis

We used spatial cluster-outlier statistical approaches to assess the geographic patterns of county-level model-based disability estimates via ArcGIS version 10.8.1 (Esri). The spatial cluster-outlier analysis is based on the Anselin Local Moran’s *I* statistic, a local indicator of spatial association (19,20). A county with a positive value for Moran’s *I* indicates that it has a similar high or low value as its neighboring counties and is a member of the same cluster, whereas a negative value of Moran’s *I* indicates that it could be a geographic outlier compared with its neighboring counties. In other words, its value is dissimilar to the values of its geographic neighbors. The cluster-outlier was considered significant if *P* < .05 for the Moran’s *I* statistic. The ArcGIS spatial analysis tool provides 4 types of indicators at the county level with 95% CIs: 1) High–high cluster, a significant cluster of a high-value county surrounded by high-value counties; 2) Low–low cluster, a significant cluster of a low-value county surrounded by low-value counties; 3) High–low outlier, a high-value county surrounded by low-value counties, which denotes that county as a high-value outlier; and 4) Low–high, a low-value county surrounded by high-value counties, which denotes that county as a low-value outlier. We calculated the percentage of counties in each urban–rural category that were in a high–high or low–low cluster.

We adopted a validation approach similar to the one used by Zhang et al (12) and Wang et al (13) and compared the model-based estimates with BRFSS direct survey estimates at the state level (internal validation). We calculated Pearson correlation coefficients to assess the correlation between the 2 sets of disability estimates, and also compared the BRFSS county-level model-based estimates with ACS 1-year direct estimates for 827 of the 3,142 counties; 2018 ACS 1-year data provides only 827 of 3,142 county-level estimates.

We mapped the 6 functional disability prevalences by using Jenks natural breaks classification and by quartiles for any disability prevalence. Jenks classifies data based on similar values and maximizes the differences between classes.

## Results

Overall, among the 3,142 counties, the estimated median prevalence was 8.0% (IQR, 7.0%–9.2%) for hearing, 4.9% (IQR, 4.1%–6.1%) for vision, 11.5% (IQR, 9.7%–13.7%) for cognition, 14.9% (IQR, 12.5%–18.0%) for mobility, 3.7% (IQR, 3.1%–4.5%) for self-care, 7.2% (IQR, 6.1%–8.5%) for independent living, and 29.5% (IQR, 25.6%–34.2%) for any disability (Table 1). When stratified by county urban–rural classification, hearing disability and any disability estimates increased by county rurality, whereas the other 5 disability types did not have such patterns.

**Table 1:** Summary of Model-Based Estimates by Disability Type and NCHS County Urban–Rural Classification Scheme, US, 2018

Disability type/county class	No. of counties	Prevalence estimate, %	
		Median (IQR)	Range^a^ (minimum–maximum)
**Hearing**			
Large central metro	68	5.7 (5.1–6.2)	5.2 (3.5–8.7)
Large fringe metro	368	6.5 (5.7–7.3)	6.4 (3.7–10.1)
Medium metro	372	7.1 (6.4–8.3)	9.0 (4.2–13.2)
Small metro	358	7.5 (6.7–8.3)	9.0 (4.8–13.8)
Micropolitan	641	7.9 (7.2–8.9)	8.4 (4.5–12.9)
Noncore	1,335	9.0 (8.0–10.0)	10.2 (5.1–15.3)
All counties	3,142	8.0 (7.0–9.2)	11.8 (3.5–15.3)
**Vision**			
Large central metro	68	5.2 (4.3–5.9)	7.0 (3.1–10.1)
Large fringe metro	368	4.1 (3.5–4.8)	6.5 (2.5–9.0)
Medium metro	372	4.8 (4.0–5.7)	9.1 (2.6–11.7)
Small metro	358	4.9 (4.1–5.8)	10.2 (2.4–12.6)
Micropolitan	641	5.1 (4.2–6.1)	12.0 (2.8–14.7)
Noncore	1,335	5.3 (4.3–6.6)	14.2 (2.9–17.2)
All counties	3,142	4.9 (4.1–6.1)	14.7 (2.4–17.2)
**Cognition**			
Large central metro	68	11.4 (10.0–12.8)	8.3 (7.5–15.8)
Large fringe metro	368	10.3 (8.9–11.9)	12.0 (6.2–18.2)
Medium metro	372	11.8 (10.0–13.3)	13.0 (7.2–20.2)
Small metro	358	11.7 (10.1–13.7)	15.4 (6.5–21.9)
Micropolitan	641	12.1 (10.3–14.1)	19.3 (6.4–25.7)
Noncore	1,335	11.5 (9.6–14.5)	22.8 (6.7–29.4)
All counties	3,142	11.5 (9.7–13.7)	23.3 (6.2–29.4)
**Mobility**			
Large central metro	68	12.8 (10.7–15.1)	12.3 (7.5–19.8)
Large fringe metro	368	12.9 (10.6–14.8)	16.7 (5.9–22.7)
Medium metro	372	14.4 (12.0–16.7)	20.9 (6.5–27.4)
Small metro	358	14.4 (11.8–16.7)	17.0 (7.9–24.9)
Micropolitan	641	15.3 (12.8–18.0)	22.2 (7.1–29.3)
Noncore	1,335	16.2 (13.2–19.5)	27.0 (7.9–34.9)
All counties	3,142	14.9 (12.5–18.0)	29.0 (5.9–34.9)
**Self-care**			
Large central metro	68	3.6 (3.1–4.1)	4.3 (2.1–6.4)
Large fringe metro	368	3.1 (2.6–3.5)	4.9 (2.0–6.9)
Medium metro	372	3.5 (3.0–4.1)	6.2 (2.1–8.3)
Small metro	358	3.6 (3.1–4.2)	6.9 (1.9–8.8)
Micropolitan	641	3.8 (3.2–4.5)	8.0 (2.2–10.2)
Noncore	1,335	4.0 (3.3–4.8)	10.3 (2.3–12.7)
All counties	3,142	3.7 (3.1–4.5)	10.8 (1.9–12.7)
**Independent living**			
Large central metro	68	6.8 (5.9–7.7)	6.4 (4.4–10.8)
Large fringe metro	368	6.2 (5.3–7.0)	7.3 (3.8–11.1)
Medium metro	372	7.0 (6.1–8.0)	9.7 (4.2–13.9)
Small metro	358	7.1 (6.2–8.2)	11.2 (3.6–14.8)
Micropolitan	641	7.5 (6.4–8.6)	13.1 (4.0–17.0)
Noncore	1,335	7.5 (6.2–9.2)	15.5 (4.3–19.8)
All counties	3,142	7.2 (6.1–8.5)	16.2 (3.6–19.8)
**Any disability**			
Large central metro	68	25.6 (22.3–28.3)	19.0 (15.5–34.5)
Large fringe metro	368	25.8 (22.1–29.7)	27.3 (12.9–40.2)
Medium metro	372	28.5 (24.9–32.5)	32.1 (15.8–47.8)
Small metro	358	29.0 (25.2–32.6)	29.3 (17.6–47.0)
Micropolitan	641	30.4 (26.5–34.9)	34.5 (15.7–50.3)
Noncore	1,335	31.3 (26.8–36.3)	37.3 (17.9–55.2)
All counties	3,142	29.5 (25.6–34.2)	42.3 (12.9–55.2)

Abbreviation: NCHS, National Center for Health Statistics.

a Difference between minimum and maximum.

**Figure 1:**
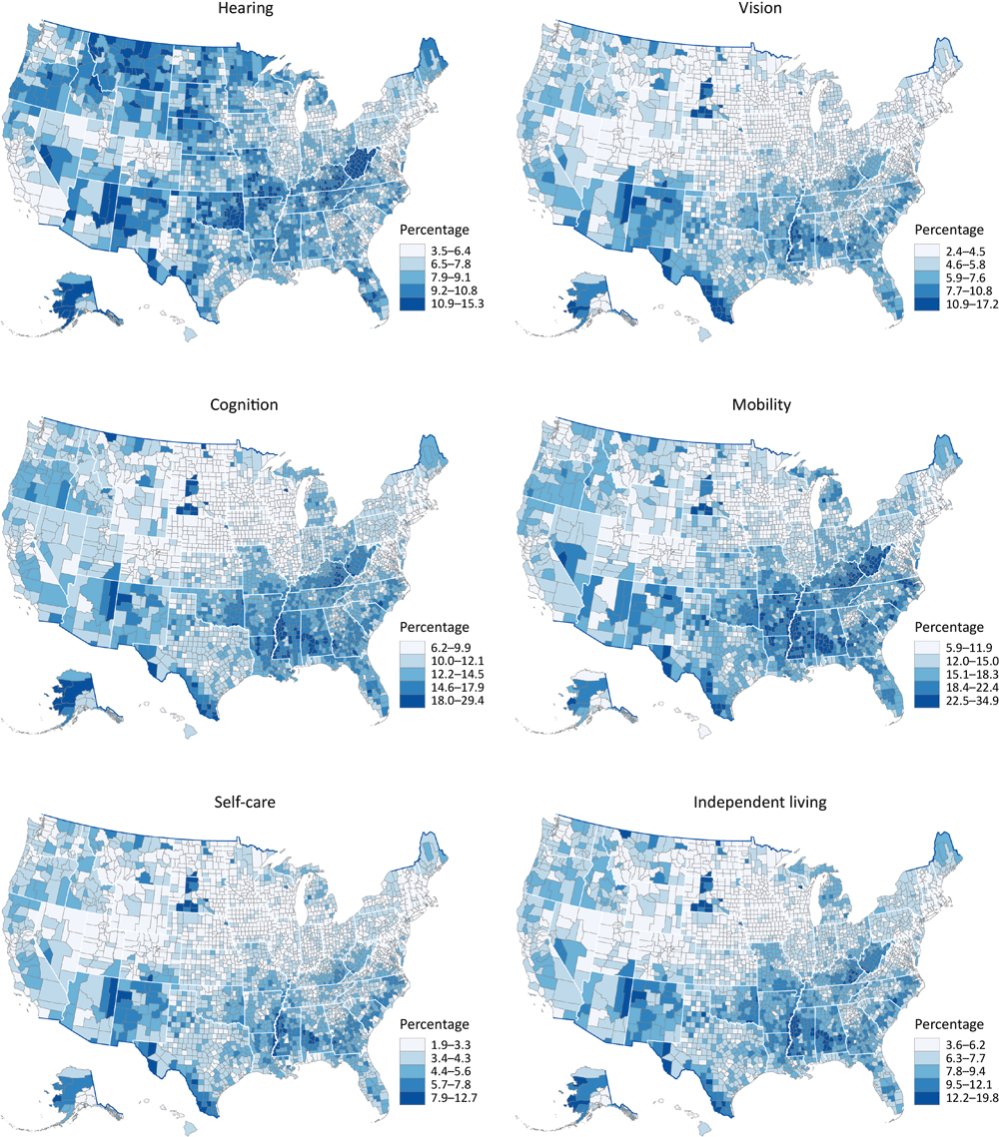
County-level model-based estimates among adults aged ≥18 years by disability type, United States, 2018. Maps were classified into 5 classes by using Jenks natural breaks. Data sources: Behavioral Risk Factor Surveillance System 2018 (10), US Census Bureau (15,16).

**Figure 2:**
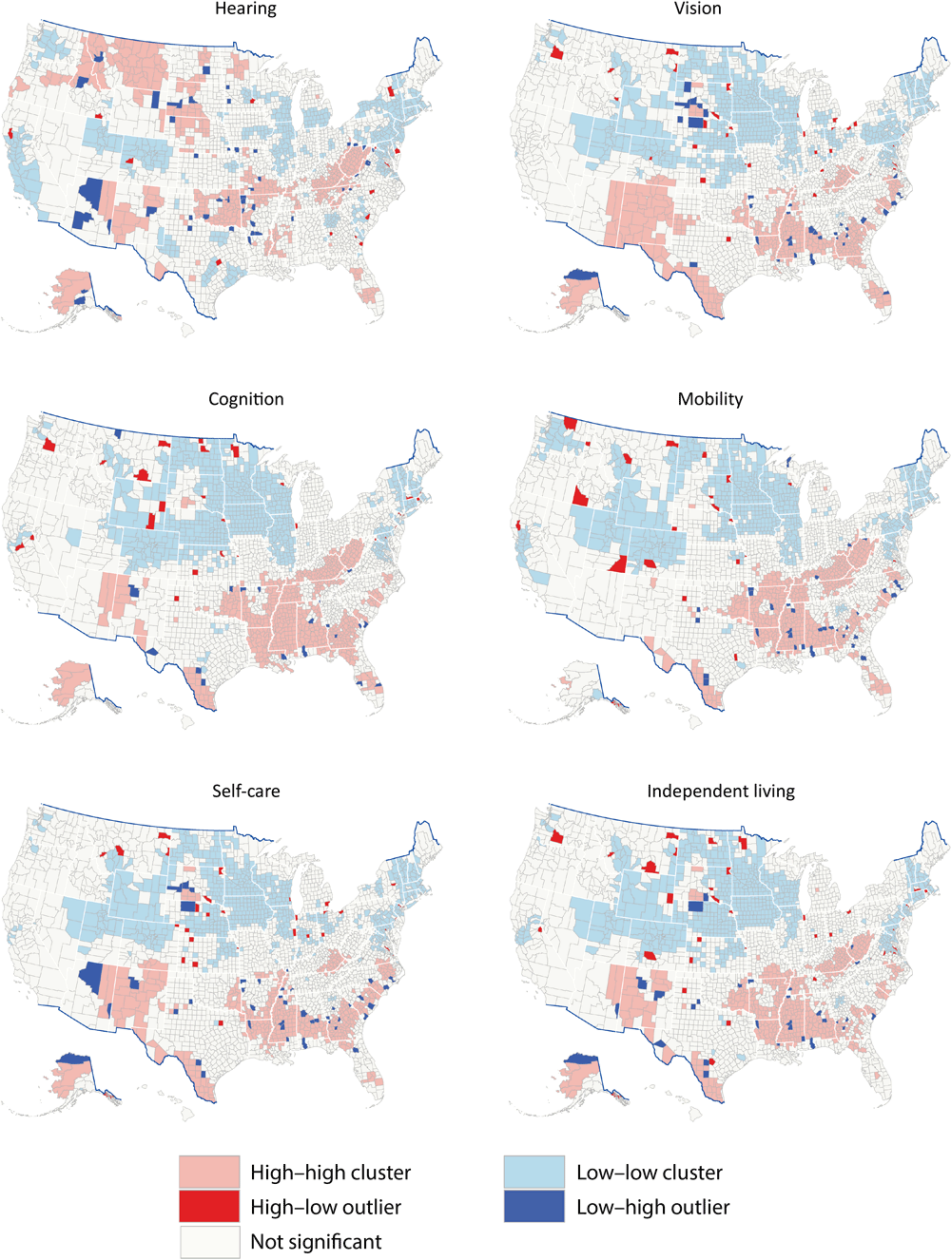
Cluster-outlier for model-based estimates among adults aged ≥18 years by functional disability type and county, United States, 2018. Data sources: Behavioral Risk Factor Surveillance System 2018 (10), US Census Bureau (15,16).

Percentages for each disability ranged as follows: for hearing, 3.5% to 15.3%; for vision, 2.4% to 17.2%; for cognition, 6.2% to 29.4%; for mobility, 5.9% to 34.9%; for self-care, 1.9% to 12.7%; and for independent living, 3.6% to 19.8% (Figure 1). Moran’s *I* was 0.62 for hearing, 0.62 for vision, 0.71 for cognition, 0.69 for mobility, 0.60 for self-care, 0.64 for independent living, and 0.70 for any disability (all *P* < .001), indicating that disability was highly clustered at the county level. For single functional disabilities (Figure 2), clusters were similar for vision, cognition, mobility, self-care, and independent living, but we observed large high–high cluster counties in New Mexico for vision and self-care and large high–high cluster counties along the southern Appalachian Mountains for cognition, mobility, and independent living. The cluster pattern for hearing differed from the other types of disability. Large high–high cluster counties for hearing were in Montana and Idaho; along the South Dakota–Nebraska border; in parts of Oklahoma, Arkansas, and Kansas; Kentucky and West Virginia; and parts of Alaska, Florida, and New Mexico.

**Figure 3:**
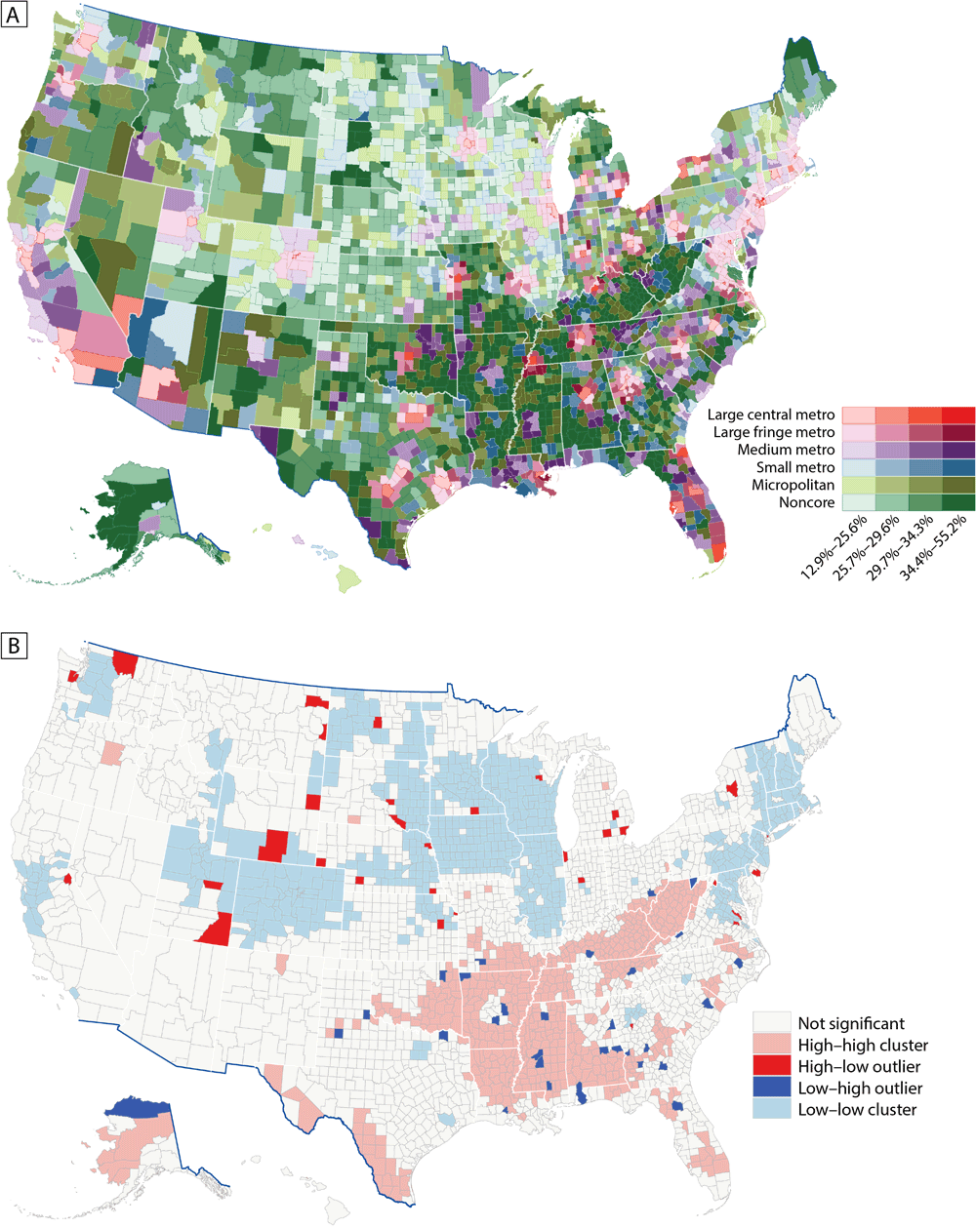
Model-based estimates of any disability among adults aged ≥18 years by county, United States, 2018. A, Prevalence by urban–rural status, classified by quartiles. B, Prevalence by cluster-outlier analysis. Data sources: Behavioral Risk Factor Surveillance System 2018 (10), US Census Bureau (15,16).

In the analysis of any disability estimates at the county level by urban–rural county classification (Figure 3), we observed a higher prevalence of any disability among counties in southern states, along the Appalachian Mountains, along the Texas–Mexico border, in New Mexico, and in Arizona (Figure 3A). For any disability, the high–high clusters included most counties in Mississippi, West Virginia, and Kentucky; all counties along the southern Mississippi River; most counties along the Texas–Mexico border; portions of Alabama, Alaska, Arkansas, Florida, rural Georgia, Louisiana, Missouri, Oklahoma, and Tennessee; and some counties in North Carolina, South Carolina, Ohio, and Virginia (Figure 3B). One large low–low cluster comprised counties in Colorado, Idaho, Utah, and Wyoming. We observed a second large low–low cluster in North Dakota, eastern South Dakota, and Nebraska; most of Iowa, Illinois, and Wisconsin; and the southern half of Minnesota. A third large low–low cluster was observed in the 6 New England states (Connecticut, Maine, Massachusetts, New Hampshire, Rhode Island, and Vermont) and the mid-Atlantic states (New Jersey and parts of New York, Pennsylvania, Maryland, and Virginia). The cluster-outlier analysis also identified counties that were outliers around high or low clusters.

In the cluster-outlier analysis (Table 2), we found that among high–high clusters, cognition had the highest percentage of counties (19.5%; 612 of 3,142) and self-care had the lowest percentage (13.6%; 428 of 3,142). Among low–low clusters, vision had the highest percentage of counties (23.3%; 731 of 3,142) and hearing had the lowest percentage (18.3%; 576 of 3,142). Compared with other types of disability, the percentage of counties in high–high clusters for hearing significantly increased from small metro (7.8%) and micropolitan (14.8%) to noncore (23.9%) and significantly decreased from large central metro (79.4%) to noncore (4.9%) in low–low clusters. For any disability, 18.9% (594 of 3,142) of counties were in high–high clusters and 22.0% (691 of 3,142) of counties were in low–low clusters. By urban–rural county classification and any disability (Table 2), noncore counties had the highest percentage of counties (24.2%; 323 of 1,335) in high–high clusters and large central metro counties had the highest percentage of counties (48.5%; 33 of 68) in low–low clusters. There were 1.1% (34 of 3,142) of counties in high–low outlier counties and 1.0% (30 of 3,142) in low–high outlier counties. Large central metro counties had the highest percentage (2.9%; 2 of 68) in high–low outliers and medium metro counties had the highest percentage in low–high outliers (3.5%; 13 of 372).

**Table 2:** Cluster-Outlier Results for Model-Based Estimates by Disability Type and County Urban–Rural Status, US, 2018

Disability type/county class	No. of counties^a^	Cluster, no. (%)		Outlier, no. (%)	
		High–high^b^	Low–low^c^	High–low^d^	Low–high^e^
**Hearing**					
Large central metro	68	0	54 (79.4)	0	1 (1.5)
Large fringe metro	368	2 (0.5)	204 (55.4)	1 (0.3)	0
Medium metro	372	35 (9.4)	98 (26.3)	2 (0.5)	7 (1.9)
Small metro	358	28 (7.8)	65 (18.2)	1 (0.3)	10 (2.8)
Micropolitan	641	95 (14.8)	90 (14.0)	2 (0.3)	16 (2.5)
Noncore	1,335	319 (23.9)	65 (4.9)	8 (0.6)	11 (0.8)
All counties	3,142	479 (15.2)	576 (18.3)	14 (0.4)	45 (1.4)
**Vision**					
Large central metro	68	0	16 (23.5)	10 (14.7)	0
Large fringe metro	368	8 (2.2)	116 (31.5)	4 (1.1)	4 (1.1)
Medium metro	372	40 (10.8)	85 (22.8)	3 (0.8)	13 (3.5)
Small metro	358	35 (9.8)	87 (24.3)	1 (0.3)	8 (2.2)
Micropolitan	641	112 (17.5)	164 (25.6)	3 (0.5)	0
Noncore	1,335	249 (18.7)	263 (19.7)	11 (0.8)	9 (0.7)
All counties	3,142	444 (14.1)	731 (23.3)	32 (1.0)	34 (1.1)
**Cognition**					
Large central metro	68	2 (2.9)	17 (25.0)	5 (7.4)	0
Large fringe metro	368	16 (4.3)	97 (26.4)	5 (1.4)	3 (0.8)
Medium metro	372	71 (19.1)	62 (16.7)	3 (0.8)	8 (2.2)
Small metro	358	66 (18.4)	72 (20.1)	2 (0.6)	3 (0.8)
Micropolitan	641	141 (22.0)	139 (21.7)	4 (0.6)	2 (0.3)
Noncore	1,335	316 (23.7)	321 (24.0)	9 (0.7)	3 (0.2)
All counties	3,142	612 (19.5)	708 (22.5)	28 (0.9)	19 (0.6)
**Mobility**					
Large central metro	68	0	28 (41.2)	2 (2.9)	0
Large fringe metro	368	9 (2.4)	116 (31.5)	2 (0.5)	1 (0.3)
Medium metro	372	51 (13.7)	75 (20.2)	2 (0.5)	14 (3.8)
Small metro	358	54 (15.1)	83 (23.2)	1 (0.3)	10 (2.8)
Micropolitan	641	136 (21.2)	141 (22.0)	2 (0.3)	4 (0.6)
Noncore	1,335	309 (23.1)	241 (18.1)	14 (1.0)	5 (0.4)
All counties	3,142	559 (17.8)	684 (21.8)	23 (0.7)	34 (1.1)
**Self-care**					
Large central metro	68	0	24 (25.3)	8 (11.8)	0
Large fringe metro	368	8 (2.2)	118 (32.1)	4 (1.1)	4 (1.1)
Medium metro	372	35 (9.4)	79 (21.2)	3 (0.8)	16 (4.3)
Small metro	358	38 (10.6)	83 (23.2)	1 (0.3)	10 (2.8)
Micropolitan	641	102 (15.9)	150 (23.4)	3 (0.5)	5 (0.8)
Noncore	1,335	245 (18.4)	231 (17.3)	14 (1.0)	7 (0.5)
All counties	3,142	428 (13.6)	685 (21.8)	33 (1.1)	42 (1.3)
**Independent living**					
Large central metro	68	0	24 (25.3)	9 (13.2)	0
Large fringe metro	368	6 (1.6)	112 (30.4)	6 (1.6)	4 (1.1)
Medium metro	372	38 (10.2)	66 (17.7)	4 (1.1)	10 (2.7)
Small metro	358	49 (13.7)	74 (20.7)	1 (0.3)	4 (1.1)
Micropolitan	641	125 (19.5)	131 (20.4)	2 (0.3)	1 (0.2)
Noncore	1,335	280 (21.0)	268 (20.1)	16 (1.2)	10 (0.7)
All counties	3,142	498 (15.8)	675 (21.5)	38 (1.2)	29 (0.9)
**Any disability**					
Large central metro	68	1 (1.5)	33 (48.5)	2 (2.9)	0
Large fringe metro	368	13 (3.5)	132 (35.9)	6 (1.6)	0
Medium metro	372	57 (15.3)	73 (19.6)	2 (0.5)	13 (3.5)
Small metro	358	55 (15.4)	83 (23.2)	4 (1.1)	9 (2.5)
Micropolitan	641	145 (22.6)	132 (20.6)	5 (0.8)	4 (0.6)
Noncore	1,335	323 (24.2)	238 (17.8)	15 (1.1)	4 (0.3)
All counties	3,142	594 (18.9)	691 (22.0)	34 (1.1)	30 (1.0)

a Number of counties in cluster or outlier.

b High-value county surrounded by high-value counties.

c Low-value county surrounded by low-values counties.

d High-value county surrounded by low value-counties.

e Low-value county surrounded by high-value counties.

The model-based estimates for all disability indicators were significantly and highly correlated with BRFSS direct estimates at the state level (Table 3). In the comparison of BRFSS county-level model-based estimates with ACS 1-year direct estimates for 827 counties, in general, BRFSS had higher estimates than the ACS. However, they were still positively related (Table 3).

**Table 3:** Comparison of BRFSS State-Level Model-Based Estimates of Disability With BRFSS State-Level Direct Estimates and Comparison of BRFSS County-Level Model-Based Estimates With ACS 1-Year County-Level Direct Estimates, US, 2018

Level and disability	Median (IQR)	Range^a^ (minimum–maximum)	Pearson correlation coefficient^b^
**State level (N = 51^c^)**			
**Hearing**			
BRFSS direct	7.0 (6.0–7.9)	11.7 (3.1–14.8)	0.96
Model-based	6.9 (6.1–7.5)	8.1 (3.5–11.6)	
**Vision**			
BRFSS direct	4.8 (4.0–6.1)	6.1 (2.9–9.0)	0.91
Model-based	4.5 (3.9–5.3)	3.8 (3.4–7.2)	
**Cognition**			
BRFSS direct	11.0 (9.7–12.9)	10.1 (7.8–17.9)	0.95
Model-based	10.9 (9.2–12.2)	7.7 (8.3–15.9)	
**Mobility**			
BRFSS direct	13.0 (11.0–15.3)	14.7 (8.5–23.3)	0.98
Model-based	12.4 (10.8–14.7)	12.4 (8.5–20.9)	
**Self-care**			
BRFSS direct	3.5 (3.0–4.3)	4.4 (2.1–6.5)	0.88
Model-based	3.3 (3.0–3.9)	2.7 (2.5–5.2)	
**Independent living**			
BRFSS direct	6.9 (5.7–7.8)	7.5 (4.9–12.4)	0.94
Model-based	6.7 (5.7–7.4)	4.7 (5.1–9.7)	
**Any disability**			
BRFSS direct	27.0 (23.9–29.7)	22.8 (19.6–42.3)	0.98
Model-based	26.0 (22.6–28.5)	21.8 (18.8–40.6)	
**County level (N = 827^d^)**			
**Hearing**			
ACS 1-year	4.7 (3.8–5.8)	9.5 (1.8–11.3)	0.65
Model-based	6.7 (5.9–7.5)	9.1 (3.5–12.6)	
**Vision**			
ACS 1-year	2.7 (2.1–3.5)	9.3 (0.8–10.1)	0.47
Model-based	4.5 (3.9–5.4)	9.8 (2.5–12.3)	
**Cognition**			
ACS 1-year	5.4 (4.5–6.6)	11.7 (1.3–13.0)	0.51
Model-based	11.2 (9.7–12.7)	14.3 (6.2–20.5)	
**Mobility**			
ACS 1-year	8.4 (6.7–10.2)	17.9 (3.4–21.3)	0.75
Model-based	13.2 (11.1–15.5)	17.4 (5.9–23.4)	
**Self-care**			
ACS 1-year	2.9 (2.3–3.6)	6.4 (0.8–7.2)	0.55
Model-based	3.4 (2.9–3.9)	6.6 (2.0–8.6)	
**Independent living**			
ACS 1-year	5.8 (4.8–7.2)	10.8 (2.2–13.0)	0.58
Model-based	6.7 (5.8–7.6)	10.8 (3.8–14.6)	
**Any disability**			
ACS 1-year	15.9 (13.2–18.8)	24.3 (7.1–31.4)	0.70
Model-based	27.0 (23.3–30.6)	30.5 (12.9–43.5)	

Abbreviations: ACS, American Community Survey; BRFSS, Behavioral Risk Factor Surveillance System.

a Difference between minimum and maximum.

b All Pearson correlation coefficients are significant at *P* < .001.

c Includes the District of Columbia.

d 2018 ACS 1-year data provide only 827 of 3,142 county-level estimates.

## Discussion

This study generated county-level estimates for 6 disability types and any disability among US adults, showing substantial geographic variations in the 6 disability types and any disability across US counties and differential variations by county urban–rural status. The spatial cluster analysis indicated that the 6 types of disability and any disability were spatially clustered at the county level. Furthermore, we observed similar spatial cluster patterns among the various disability types, except for hearing disability. Hearing disability mostly clustered in Idaho, Montana and Wyoming, the West North Central states, and along the Appalachian Mountains.

A previous report indicated that, nationwide, adults living in nonmetropolitan counties had a higher prevalence of the 6 types of disability or any disability than did those living in metropolitan counties (21). Our results further presented estimates of disabilities distribution at the county level and clusters among 6 urban–rural county levels. We found prevalence by county rurality increased for hearing disability and any disability only; and noncore counties were more likely than other counties to be in high–high clusters for hearing disability. For the states with most counties in low–low value clusters, further investigation is needed of the high-value outlier.

Multiple reasons exist for spatial variation and spatial cluster patterns of these county-level prevalences of disabilities. Patterns might reflect the spatial clusters of population characteristics such as higher percentages of older adults (aged ≥65 years), particular racial or ethnic groups, adults living below the federal poverty level, or other factors that may be related to disability. For example, counties in southern states that had a higher prevalence of obesity (22) or larger proportion of the population living below the federal poverty level (23) were more likely than counties in other states to be in high–high clusters with a higher prevalence of disabilities for vision, cognition, mobility, self-care, and independent living. The different cluster patterns for hearing might be partly attributed to industries in these geographic areas and occupational hearing loss. For example, people working in agriculture, forestry, logging, manufacturing, mining, and oil and gas drilling can be exposed to prolonged or excessive noise that may lead to hearing loss (24). In addition, hearing loss was more likely to be reported among men, non-Hispanic American Indian or Alaska Native adults, and non-Hispanic White adults (25) than among other races and ethnicities. Further investigation that uses data sources other than those we used is needed to examine the underlying population and type of industries in those areas. Health behaviors such as higher rates of smoking (26,27) and obesity (28,29) may be associated with social and environmental factors, such as quality of education, access to health care (4), access to opportunities to engage in an active lifestyle, and access to fresh and healthy food. Further investigation is needed to explore concentrations of characteristics (eg, social, familial, occupational) that may contribute to hearing disability prevalence in high-high cluster areas.

The utility of the MRP approach for providing reliable model-based small-area estimates for public health planning was previously assessed (12–14). Our study showed that small-area estimation results using the MRP method were again well correlated with the state-level survey data. The county-level modeled estimates were moderately correlated with ACS estimates, which is typical in small-area estimation validation because of differences in survey design, sampling, weighting, questionnaire, data collection model, report bias, nonresponse bias, and other differences (30). BRFSS provides the opportunity to estimate annual county-level disability by health risk behaviors, chronic conditions, health care access, and health status that is not possible by using ACS data (1). Because of numerous methodologic differences, it is difficult to directly compare BRFSS and ACS data. However, both provide useful and complementary information for assessing the health needs of people with disabilities. Further examination using ACS data of county-level variation is warranted.

Several limitations should be noted. First, the potential recall and reporting biases during BRFSS data collection remained in the model-based estimates. Second, the county population estimates used for poststratification were not census counts and thus, were subject to inaccuracy. Third, the models that we constructed did not account for policy and programs for people with disabilities at local levels due to the lack of such information. Despite these limitations, the results can be used as a starting point to better understand the local-level disparities of disabilities and help guide interventions or allocate health care service resources to the areas with the greatest need.

The findings in this study may help inform local areas on where to implement policy and programs to improve the life of people with disabilities, for example, including people with disabilities in public health programs and activities such as providing educational activities on promoting a healthy lifestyle (eg, physical activity, healthy foods), and reducing tobacco, alcohol, or drug use (31); implementing policies for addressing accessibility in physical and digital environments; and developing programs and practices that consider the needs and preferences of people with disabilities.

Our findings highlight geographic differences and clusters of disability across US counties, which can provide useful information for state and local policy makers and disability service providers to assess allocation of public health resources and to implement evidence-based intervention programs to improve health outcomes and quality of life for people living with a disability in the United States.
